# Supplementary material for: Relationship between Photosynthetic CO2 Assimilation and Chlorophyll Fluorescence for Winter Wheat under Water Stress
Source: Plants (Basel). 2023 Sep 23;12(19):3365. doi: 10.3390/plants12193365 (PMC10574178; doi:10.3390/plants12193365)
Supplement: Supplementary file 1 [file plants-12-03365-s001.zip › plants-2611670-supplementary.pdf]

Supporting Information

Relationship between photosynthetic CO_2 assimilation and chlorophyll fluorescence for winter wheat under water stress

Qianlan Jia[1], Zhunqiao Liu[2], Chenhui Guo[1], Yakai Wang[1], Jingjing Yang[3,4], Qiang Yu[2,5], Jing Wang[2], Fenli Zheng[2,4], Xiaoliang Lu[2*]

[1]College of Natural Resources and Environment, Northwest A&F University, Yangling, Shaanxi 712100, China

[2]State Key Laboratory of Soil Erosion and Dryland Farming on the Loess Plateau, Northwest A&F University, Yangling, Shaanxi 712100, China

[3]The Research Center of Soil and Water Conservation and Ecological Environment, Chinese Academy of Sciences and Ministry of Education, Yangling, Shaanxi 712100, China

[4]Institute of Soil and Water Conservation, Chinese Academy of Sciences and Ministry of Water Resources, Yangling, Shaanxi 712100, China

[5]College of Resources and Environment, University of Chinese Academy of Sciences, Beijing 100049, China

*Corresponding to: luxiaoliang@nwafu.edu.cn

Notes S1. Calculation of Φ_P, Φ_F, Φ_N, Φ_D, NPQ, Φ_{Pmax} and J_{a_PAM}

Note that NPQ, Φ_P and J_{a_PAM} are defined as (obtained from the PAM measurement):

$$NPQ = \frac{(F_m - F_I) - (F_m' - F_I)}{(F_m' - F_I)}$$

$$\Phi_P = \frac{(F_m' - F_I) - (F_t - F_I)}{(F_m' - F_I)}$$

$$J_{a\_PAM} = PAR \times \Phi_P \times 0.84 \times 0.5$$

where F_m is the maximal fluorescence in the dark; F_m' is the maximal fluorescence chlorophyll yield from light-adapted state; F_t is the steady-state fluorescence emission; F_I is the PSI fluorescence yield; PAR is photochemically active radiation. The fluorescence yields (F_o, F_m, F_m', F_t) were directly measured by the PAM fluorometer.

We have (van der Tol *et al.*, 2014):

$$K_N = \left(\frac{(F_m - F_I) - (F_m' - F_I)}{(F_m' - F_I)}\right) \times (K_F + K_D)$$

$$K_P = \left(\frac{(F_m' - F_I) - (F_t - F_I)}{(F_t - F_I)}\right) \times (K_N + K_F + K_D)$$

where K_N and K_P are the rate coefficients of energy-dependent heat dissipation and photochemistry, respectively (van der Tol *et al.*, 2014); K_F and K_D are the rate constant for constitutive heat loss and fluorescence emission (van der Tol *et al.*, 2014). In this study, K_D and K_F are assumed to be 0.9 and 0.1, respectively (Liu *et al.*, 2022). Note that NPQ should be equal to K_N because NPQ = $K_N/(K_F + K_D)$, and $K_F + K_D = 1$.

Thus:

$$\Phi_P = \frac{K_P}{K_D + K_F + K_N + K_P}$$

$$\Phi_F = \frac{K_F}{K_D + K_F + K_N + K_P}$$

$$\Phi_N = \frac{K_N}{K_D + K_F + K_N + K_P}$$

$$\Phi_D = \frac{K_D}{K_D + K_F + K_N + K_P}$$

where the quantum yields (Φ) for the four different pathways: fluorescence (Φ_F), photosynthesis (Φ_P), regulated heat dissipation (Φ_N), and basal heat dissipation (Φ_D) are obtained from the PAM measurement. It is noteworthy that Φ_P, Φ_F, Φ_N and Φ_D were also corrected for PSI fluorescence.

Figure S1. Variations in β_S and β_B for the water stress (WS, diamonds) treatments under 12 days of progressive drought stress. The red diamonds represent β_S in the WS treatment, and the black diamonds indicate β_B in the WS treatment. β_S and β_B are the normalized soil moisture dependent stress functions describing the effect of water stress in the modified Ball–Woodrow–Berry model.

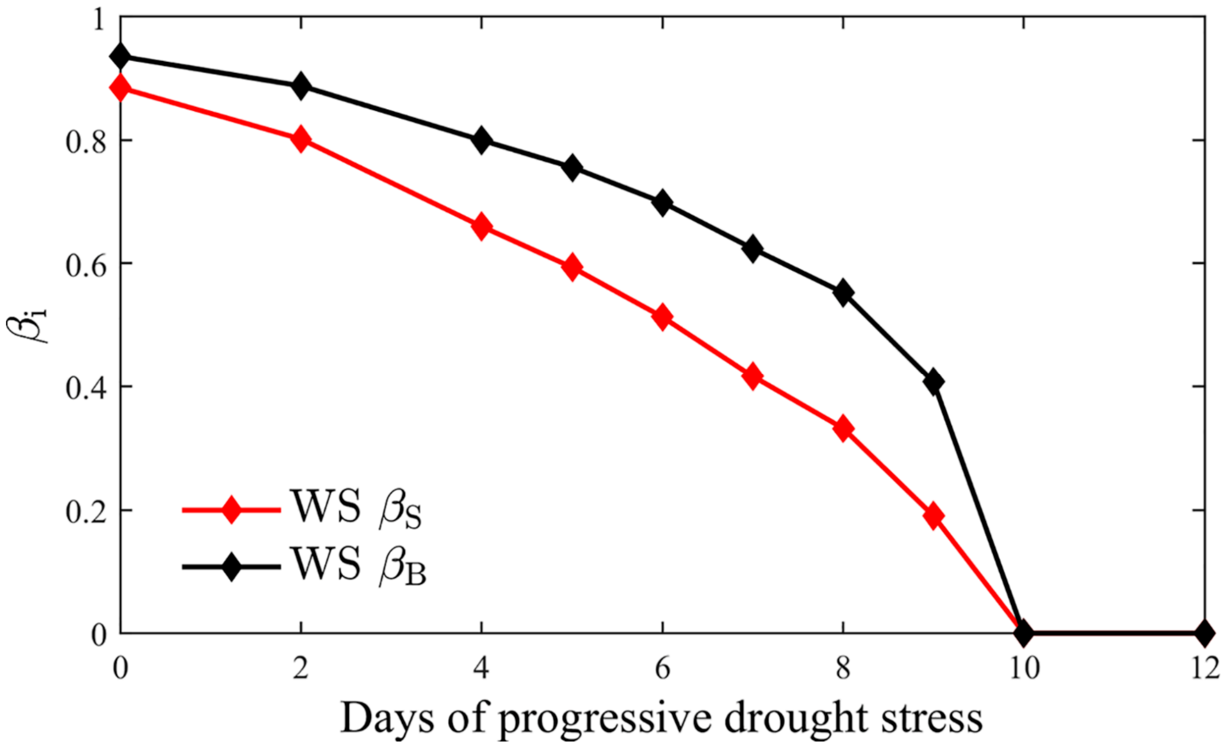

Figure S2. Average absorbed photosynthetically active radiation (APAR, µmol m^{-2} s^{-1}) under changing light intensity over the duration of the drought. The numbers in the upper right corner of each plot are the number of days after withholding water. The soil water content (SWC, %) is also indicated in each panel. The solid lines represent the mean, and the shaded areas ± one standard deviation.

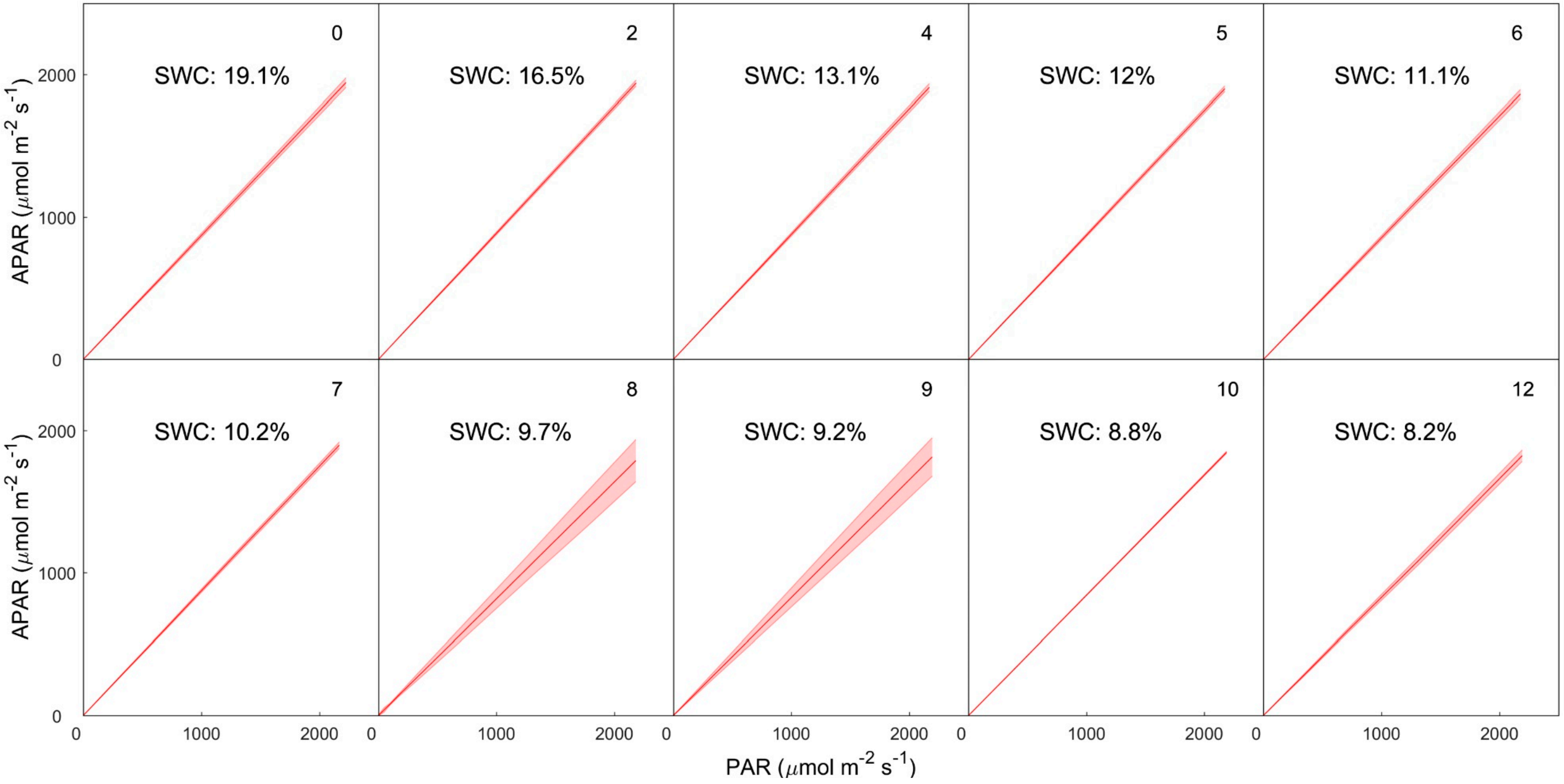

Figure S3. The responses of full-band chlorophyll fluorescence emission at the photosystem level ($ChlF_{P\_F}$, μmol m^{-2} s^{-1}) to changing light intensity during the period between 14 and 28 days (WS14 to WS28) after withholding water. Numbers in the upper right corner represent days of progressive drought stress. The soil water content (SWC, %) is also given in each panel. The solid lines represent the mean, and the shaded areas ± one standard deviation, of four replicates.

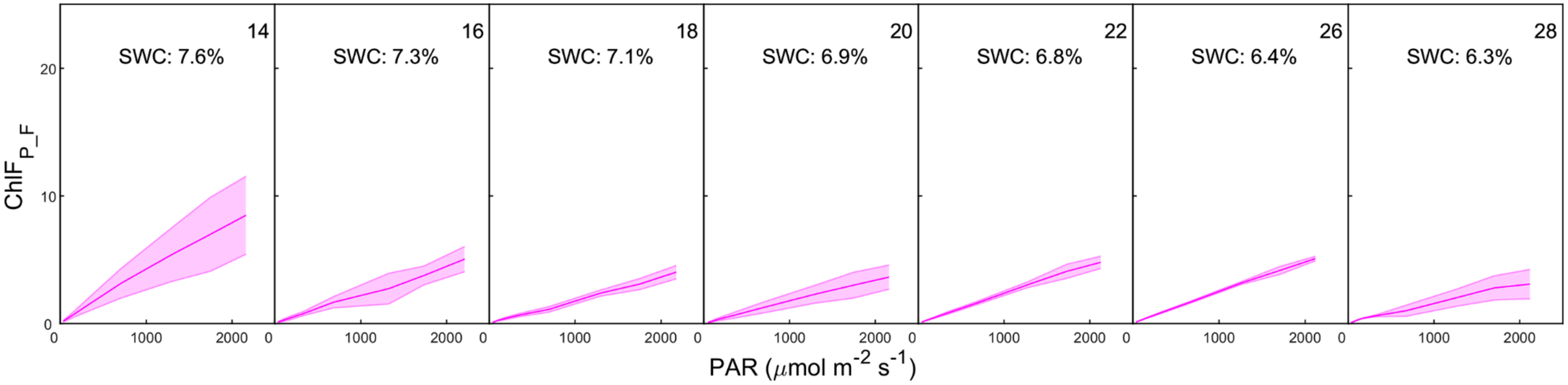

Table S1. List of retrieved parameters, their initial values, lower boundaries (LB), upper boundaries (UB) and constraints.

	Initial values	LB	UB	Constraints
g	-22030	-500000	0	NPQ
h	1.142	0	5	NPQ
j	2.539	0	5	NPQ
k	-88.11	-100	0	Φ_P
l	1.027	0	5	Φ_P
m	0.489	0	5	Φ_P
q_B	0.5	0	3	V_{cmax}
q_S	0.5	0	3	G_S

Supplemental reference:

Liu ZQ, Zhao F, Liu XJ, Yu Q, Wang YF, Peng XB, Cai HJ, Lu XL. 2022. Direct estimation of photosynthetic CO_2 assimilation from solar-induced chlorophyll fluorescence (SIF). Remote Sensing of Environment 271, 112893.

van der Tol C, Berry J, Campbell P, Rascher U. 2014. Models of fluorescence and photosynthesis for interpreting measurements of solar-induced chlorophyll fluorescence. Journal of Geophysical Research: Biogeosciences 119, 2312-2327.
